# Patient-Specific iPSCs-Based Models of Neurodegenerative Diseases: Focus on Aberrant Calcium Signaling

**DOI:** 10.3390/ijms23020624

**Published:** 2022-01-06

**Authors:** Dmitriy A. Grekhnev, Elena V. Kaznacheyeva, Vladimir A. Vigont

**Affiliations:** Laboratory of Ionic Channels of Cell Membranes, Department of Molecular Physiology of the Cell, Institute of Cytology, Russian Academy of Sciences, 4 Tikhoretsky Ave., 194064 St. Petersburg, Russia; dima.grehnyov@yandex.ru (D.A.G.); evkazn@incras.ru (E.V.K.)

**Keywords:** patient-specific models, calcium signaling, Alzheimer’s disease, Parkinson’s disease, Huntington’s disease, spinocerebellar ataxia, amyotrophic lateral sclerosis, induced pluripotent stem cells, iPSCs, neurodegeneration

## Abstract

The development of cell reprogramming technologies became a breakthrough in the creation of new models of human diseases, including neurodegenerative pathologies. The iPSCs-based models allow for the studying of both hereditary and sporadic cases of pathologies and produce deep insight into the molecular mechanisms underlying neurodegeneration. The use of the cells most vulnerable to a particular pathology makes it possible to identify specific pathological mechanisms and greatly facilitates the task of selecting the most effective drugs. To date, a large number of studies on patient-specific models of neurodegenerative diseases has been accumulated. In this review, we focused on the alterations of such a ubiquitous and important intracellular regulatory pathway as calcium signaling. Here, we reviewed and analyzed the data obtained from iPSCs-based models of different neurodegenerative disorders that demonstrated aberrant calcium signaling.

## 1. Introduction

Neurodegenerative diseases are one of the most socially significant problems facing modern medicine. The number of people suffering from neurodegenerative pathologies is growing every year. According to data from the World Health Organization, more than 55 million people live with dementia (predominantly Alzheimer’s disease) worldwide, and there are nearly 10 million new cases every year. As the proportion of older people in the population is increasing in most countries, this number is expected to rise to 78 million in 2030 and 139 million in 2050 [1]. Parkinson’s disease (PD) is the second most frequent neurodegenerative pathology. Over recent decades, the number of people suffering from PD has more than doubled to over 6 million. Of all the neurological disorders, PD has increased at the fastest rate [2]. The progression of dementia leads to the disability of patients and requires colossal expenses for their medical support. Thus, the development of new approaches to treating neurodegenerative diseases remains highly relevant. Unfortunately, most of the existing methods of therapy are aimed at symptomatic treatment and do not address the basic causes of the pathologies. Therefore, one of the most important tasks of modern neuroscience is to study the molecular mechanisms underlying neurodegeneration and search for new targets for the treatment of neurodegenerative pathologies. To solve this important issue, the development of adequate models reflecting, as accurately as possible, the pathological processes of a particular disease is required. The discovery of induced pluripotency [3] and the improvement of protocols for stem cell differentiation was a real breakthrough along this path. The main advantages of models based on patient-specific induced pluripotent stem cells (iPSCs) are the endogenous expression of mutant genes and the possibility of studying the sporadic cases of diseases. Moreover, the targeted differentiation into different types of neurons including highly vulnerable and commonly protected neurons makes it possible to study the molecular basis of selective neuronal death.

The previously proposed calcium hypothesis of neurodegeneration [4] postulates that the main factor triggering neurodegenerative processes in neurons is a disturbance of intracellular calcium signaling. Indeed, impaired calcium homeostasis has been reported for a wide range of neurodegenerative diseases, in both cellular and animal model studies [5,6,7,8,9,10]. In this review, we have summarized the data accumulated to date on established iPSCs-based models of various neurodegenerative pathologies, focusing on the revealed changes in calcium signaling.

## 2. Problems and Prospects of iPSCs-Based Models of Neurodegenerative Diseases

The era of cell differentiation began with the production and culturing of embryonic stem cells (ESCs) [11,12]. These cells have pluripotent properties and can be differentiated into any type of adult cells. With the development of differentiation protocols, the use of ESCs has become widespread for modeling neurodegenerative pathologies, including Alzheimer’s disease (AD) [13,14,15], Parkinson’s disease (PD) [16,17,18], Huntington’s disease (HD) [19,20], spinocerebellar ataxias (SCA) [21], amyotrophic lateral sclerosis (ALS) [22,23,24], and other neurological disorders [25]. However, the use of human ESCs is limited by access to preimplantation embryos and the associated ethical issues. Therefore, to model pathologies recently, ESCs of model animals are mainly used.

**Figure 1:**
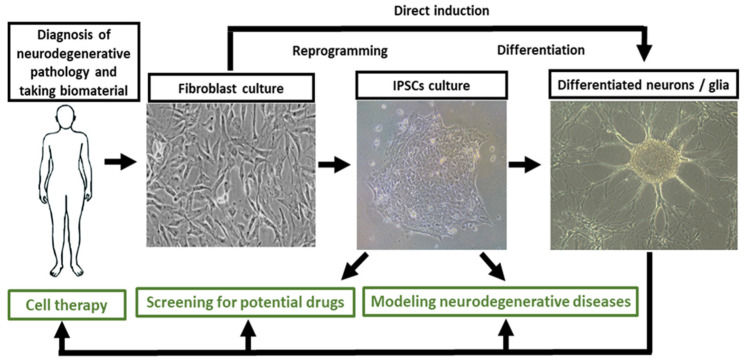
iPSCs in modeling neurodegenerative diseases, drug screening, and cell therapy.

The discovery of induced pluripotency by Professor Yamanaka created a new impulse in disease modeling using genetic reprogramming of the cells. It was postulated that the expression of only four transcription factors (Oct-4, Sox2, c-Myc, Klf4) can return a differentiated cell to the pluripotent state [3]. Those cells were called iPSCs. Numerous studies showed that iPSCs are fundamentally no different from ESCs: they can be maintained in culture and can potentially be differentiated into any type of somatic cell. However, some differences can be observed in the pattern of DNA methylation and gene expression [26,27,28]. An important advantage of iPSCs has been the elimination of many ethical problems associated with obtaining ESCs. The main applications of human iPSCs are the establishing of cellular models of diseases and also regenerative medicine and drug testing (Figure 1).

Model iPSCs lines obtained through the genetic reprogramming of a patient’s biological material are called patient-specific models. They reflect the pathological phenotype of a patient with this disease. The key advantage of iPSCs-based models is the endogenous expression of mutant genes, which makes it possible to assess the expression levels of affected genes, as well as to study the pathophysiological mechanisms under “native” conditions. Another important advantage of iPSCs-based models is the possibility of iPSCs to be differentiated into the cell type that is most vulnerable in this particular disease. In particular, it can be expected that the use of iPSCs-based models will shed light on one of the key problems of neurodegeneration—the causes of selective neuronal death.

In medical practice, the use of iPSCs creates tremendous opportunities for personalized therapy, as for each person, iPSCs from their own differentiated cells can be obtained. Subsequent genetic correction of the mutant allele and differentiation of iPSCs into cell preparations will allow for transplantation treatment. However, this approach has serious limitations due to the increased risk of tumor formation even in the case of autologous transplantation. Nevertheless, the developed cerebral organoids represent a promising transplantation source. Transplanted rat cerebral organoids demonstrate multilineage differentiation and migration in different damaged brain areas [29]. At the same time, another application of iPSCs as a platform for screening potential drugs remains highly relevant. Also, the recent advances in applying the use of high-throughput screening, computer-aided drug design, and the development of microfluidic blood–brain barrier models can significantly improve drug selection efficiency and complement iPSCs-based drug screening systems [30,31,32,33,34,35].

Drug development is very expensive but approximately 90% of drugs in human clinical trials are discarded and never sold. The main reasons for this high failure rate are lack of efficacy and existing limitations in human disease models and drug safety testing. The iPSCs-based models provide a unique opportunity for drug discovery and testing, as they enable the considering of cellular and, in particular, neuronal specificity while the activity of potential drug compounds is tested. This is highly important for the screening of neuroprotective compounds because neurodegenerative pathologies are often characterized by selective neuronal death. For example, the predominant death of striatal medium spiny neurons was detected in Huntington’s disease, whereas the most vulnerable neuronal types in Parkinson’s disease and amyotrophic lateral sclerosis are dopaminergic neurons and motor neurons, consequently. Thus, testing a drug on neurons without considering the specificity of neuronal vulnerability can significantly limit the search for effective compounds. It is expected that small molecules selected using iPSCs-based models will demonstrate similar efficacy in the treatment of patients. During the screening of chemicals, in addition to their effectiveness, their toxicity is checked. Appropriate screening using iPSCs-based models to select highly potent, low toxicity chemicals would significantly reduce drug development costs.

Despite the many advantages of using patient-specific models, challenges exist. First is the problem of genetic heterogeneity between different patients and between control and disease. Significant variations in the pathological phenotype are often observed even for patients with monogenic diseases with the same mutation. Therefore, it is important to consider the influence of other genes. To overcome this limitation, isogenic cell lines were established, differing only by a mutation in a particular gene [36,37,38,39,40,41,42,43,44,45].

Another important limitation is the fact that in vitro differentiation is different from the physiological maturation of cells in the organism. Therefore, although the iPSCs-based models can be considered among the most successful and physiologically adequate models of neurodegenerative pathologies, scientists strive to make these models even more relevant, developing, in particular, strategies for three-dimensional cultivation of cell structures similar to a primitive brain [46,47,48,49].

## 3. Obtaining the iPSCs-Based Models of Neurodegenerative Diseases

The creation of a patient-specific iPSCs-based model of the disease begins with the search for a patient with the corresponding pathology. Medical neurological centers diagnose the disease and collect biological material from donors (skin biopsy or peripheric blood sampling), who have previously signed an informed consent. Further, a fibroblast culture is obtained from the collected biological material of the patient. The next step is the return of cells to the pluripotent state (reprogramming of the fibroblast culture into iPSCs). Some models stop at this stage [50]. For the other models, the final stage is a differentiation of iPSCs into the suitable cell type, as a rule, into the most vulnerable in this pathology (Figure 1). It is important to find out at what stage of differentiation the pathological phenotype is reproduced. It has been shown that terminally differentiated cells more fully reflect the disease phenotype than do iPSCs or NPCs (neuronal progenitor cells) for many neurodegenerative pathologies [41,51,52].

The patient-specific models can be obtained by both reprogramming cells in iPSCs and their subsequent differentiation, and direct induction of somatic cells (Figure 1). Direct induction can be induced by overexpression of a set of transcription factors that promote chromatin remodeling and trigger direct cell differentiation, bypassing the stage of iPSCs generation [53,54,55,56,57].

Cell reprogramming into iPSCs is triggered by transcription factors that return a differentiated cell to a pluripotent state. To deliver genes encoding these factors into cells, the retroviruses (in particular, lentivirus) can be used to integrate the required genes into the host genome [8,58], and Sendai (non-integrative) viruses or transient transfection [59,60]. Both methods of reprogramming have similar efficiency, and the cell models obtained by these methods, as a rule, do not have any morphological or functional differences [61]. The use of the Sendai virus looks more promising, as the virus plasmid does not integrate into the cell genome, removing the risk of protooncogene activation, which may occur during an integrative lentiviral infection.

The differentiation process includes three principal stages: neuronal induction, the production of cells of the lateral ganglionic eminence (LGE), and terminal differentiation. Obtaining the required type of neurons is necessary to choosing the appropriate combination (and concentration) of cell proliferation and differentiation factors that best suits the conditions in the developing brain.

The process of establishing the iPSCs-based models of the diseases at all stages is accompanied by the careful characterization of the obtained cells. This includes confirmation of the presence or absence of specific markers, testing the ability of iPSCs to differentiate into cells of all three germ layers, and testing for teratoma formation. Karyotyping is performed to control chromosomal rearrangements that periodically occur during reprogramming and cell culture. Using the methods of immunocytochemistry and PCR, the expression of specific protein markers is analyzed: pluripotency (TRA-1-60, TRA-1-81, SSEA-3, SSEA4, Oct-4), germ layers (PAX6, SOX1 (ectoderm), GATA4, α-SMA (mesoderm), SOX17, AFP (endoderm), and differentiated cells such as neurons (MAP2, NCAM, Tuj1, DCX). In addition, morphological and functional control of the resulting cells is required. Functional confirmation of successful neuronal differentiation can include a demonstration of the ability to respond to depolarization of the plasma membrane, generation of action potentials, and forming synapses and specific morphological structures characterizing the particular neuronal type (such as spines for medium spiny neurons). When hereditary pathology is modeled, it is also necessary to confirm the presence of the mutation.

Since the first publication in 2007, describing a protocol for generating iPSCs from human somatic cells, there has been an explosion in methods of differentiation into various types of cells, including neurons. Protocols for differentiation into multiple subtypes of neurons [58,62,63,64], as well as astrocytes [65,66], microglia [67,68,69], oligodendrocytes [70], and endothelial cells [71,72], have been developed. Improved differentiation protocols continue to increase the yield, purity, and maturity of cells.

To date, it is known that various neurodegenerative diseases, including AD, PD, and HD, are characterized by impairments in different types of cells (neurons, astrocytes, oligodendrocytes, microglia, pericytes, and endothelial cells); therefore, three-dimensional (3D) and co-culture models are required to reproduce those pathology mechanisms that cannot be detected in monocultures or two-dimensional cultures [36,69,73]. This is quite important because the expression of key genes and proteins in glial cells has been proven to be varied between 2D and 3D cultures [29].

The existing iPSCs-based models already represent an important instrument for basic science studies and the search for new drugs. However, these models must be improved to obtain more precise modeling of the pathogenesis of the diseases.

## 4. Neurodegeneration and Calcium Signaling

Calcium is one of the most ubiquitous secondary messengers in the cell. Calcium signaling controls a wide range of important intracellular processes such as gene expression, proliferation, and apoptosis. The key “players” that regulate the concentration of free calcium ions in the cell include the endoplasmic (sarcoplasmic) reticulum [74], mitochondria [74,75], lysosomes [76], calcium-binding proteins [77], and ion transporters: calcium-permeable ion channels [74,78], calcium ATPases [79,80], and exchangers [81,82]. It should also be noted that mitochondrial dysfunction is closely related to aberrant calcium signaling, as mitochondria are stores of calcium in cells, and excessive calcium uptake can lead to mitochondria overload, caspase activation, and apoptosis. The major organelles that maintain calcium homeostasis in cell mitochondria and the ER also play a critical role in oxidative stress [74,75], neuroinflammation [83,84], and apoptosis [84]. Thus, alterations in the production of reactive oxygen species that can lead to ER stress should also be addressed in studies of aberrant calcium signaling.

Disturbances in various components of calcium signaling are widely spread in different pathologies, including neurodegenerative diseases [85,86,87,88]. For the first time, the calcium hypothesis of neurodegeneration was proposed by neuroscientist Zaven Khachaturian in 1982 and published in 1984 [4]. The author noted the correlation between the disturbance of calcium homeostasis in the aging brain and the occurrence of age-related neurodegenerative pathologies such as Alzheimer’s disease. In 1994, an updated version of the calcium hypothesis “Calcium hypothesis of Alzheimer’s disease and brain aging” was published, clarifying the molecular mechanisms of calcium homeostasis disturbance [89]. Later, in 2004, the calcium hypothesis for Huntington’s disease was formulated [90]. In 2009, Professor Bezprozvanny summarized the results demonstrating impaired calcium signaling in AD, PD, HD, ALS, and SCAs [85]. Nevertheless, the lack of specific drugs with proven therapeutic effects, including calcium signaling modulators [91], makes the search for new targets and compounds highly relevant. This requires further study of the molecular mechanisms of neurodegeneration, within both the framework of the calcium hypothesis and its alternatives.

To date, a great deal of data has been accumulated on disturbances in calcium signaling in different models of neurodegenerative disorders including animal and cellular models. In the last decade, the patient-specific models of neurodegenerative diseases have created new opportunities in studying the pathogenesis of these pathologies and clarifying the calcium hypothesis of neurodegeneration. Here, we systematized the data on pathological calcium signaling in iPSCs-based models of neurodegenerative diseases.

## 5. Alzheimer’s Disease

Alzheimer’s disease (AD) is one of the most widespread neurodegenerative disorders, characterized by the predominant death of neurons in the hippocampus, cerebral cortex, and locus coeruleus [92,93,94,95]. The neuropathological hallmarks of AD are amyloid aggregates, hyperphosphorylation of the tau protein, and neurofibrillary tangles [92,96]. Hereditary AD cases are caused mostly by mutations in the genes *PSEN1* and *PSEN2*, encoding presenilin proteins, and *APP*, encoding the β-amyloid precursor protein.

The first iPSCs-based AD neuronal model was performed in 2011 [97]. Further, the isogenic models were created [37,38,39,98]. Currently studied AD cellular models are 3D iPSCs-based neurons [46,48,99] and 2D cell cultures [37,97,100,101,102,103,104] including cortical neurons [105,106] and cholinergic neurons [107] as well as astrocytes [105,108], microglia [67,69], and endothelial cells [72,109] (Table 1).

In iPSCs-based models of familiar AD, an increase in the production of reactive oxygen species, impaired energy status and mitochondrial potential, lysosomal acidification, impaired autophagy and mitophagy, and aberrant calcium signaling have been shown [99,104]. The release of calcium by mitochondria and the endoplasmic reticulum plays an important role in the transmission of calcium signals in various cells. In *PSEN1*-associated AD, these pathways are disrupted, which may cause a further imbalance in calcium homeostasis (and contribute to AD pathogenesis). In particular, an increase in the rate of calcium leakage from the ER in astrocytes modeling AD has been shown [108]. It has also been reported that calcium release from the ER is enhanced in patient-specific AD neurons [104]. Moreover, inhibition of the key mitochondrial enzymatic complex alpha-ketoglutarate dehydrogenase resulted in the restoration of calcium reserves in the ER. These effects were observed in terminally differentiated neurons and were absent in iPSCs and NPCs [104].

In addition to hereditary forms of AD, genetic reprogramming technologies make it possible to study sporadic cases of the disease. In iPSCs-based models of sporadic AD, a mitochondrial dysfunction and an increase in both the production of reactive oxygen species and the number of complexes of the respiratory chain have been shown [103].

AD-specific astrocytes were also characterized by the occurrence of stress in the endoplasmic reticulum [105] and increased production of reactive oxygen species [105,108]. Also, astrocytes form the glymphatic pathway, which plays an important role in AD [110,111,112,113]. It is a waste clearance system that utilizes a unique system of perivascular channels, formed by astroglial cells, to promote the efficient elimination of soluble proteins and metabolites from the central nervous system. Intriguing data demonstrating that targeting calcium–calmodulin signaling mechanisms in astrocytes is a viable therapeutic option was obtained by Dr. Kitchen [114]. That study showed that pharmacological inhibition of calcium–calmodulin signaling events prevented the development of CNS edema and promoted functional recovery in injured rats. This role has been recently confirmed by work demonstrating that astrocytes are a viable therapeutic target using a photothrombotic stroke model [115].

## 6. Parkinson’s Disease

Parkinson’s disease (PD) is the second most common neurodegenerative disorder. The main pathophysiological feature of PD is the progressive death of dopaminergic neurons in the substantia nigra [116]. Pathological mechanisms of PD (Table 2) were studied using many iPSCs-based models, including neuroepithelial stem cells (NESC) [117], dopaminergic neurons [51,118,119,120,121,122,123,124,125,126,127,128,129,130,131,132,133,134,135,136], cortical neurons [137], and astrocytes [66] and microglia [68]. Isogenic models of PD are also widely used [41,43,45]. iPSCs-based models were obtained mainly from patients carrying mutations in genes encoding α-synuclein (*SNCA*), leucine repeat-rich kinase 2 (*LRRK2*), PTEN-induced kinase 1 (*PINK1*), ubiquitin ligase (E3) parkin (*PARK2*), a regulator of intracellular protein sorting (*VPS35*), and β-glucocerebrosidase (*GBA*).

In 1997, mutations in the *SNCA* gene were first identified as a trigger contributing to the manifestation of PD. Transcriptomic analysis of patient-specific dopaminergic neurons with mutations in *SNCA* showed impaired expression of genes associated with mitochondrial functioning [135]. Mitochondrial dysfunction not only has been identified on the gene level but also confirmed by functional assessment [43,119,131,137]. Morphological changes and decreased mitochondrial membrane potential were demonstrated [135,139]. Mutant α-synuclein disrupts the association of the endoplasmic reticulum and mitochondria, which leads to endoplasmic reticulum stress and impaired calcium homeostasis and ATP production [130,135].

Mutations in the *LRRK2* gene are among the most frequent causes of hereditary PD [141]. The most common G2019S mutation in *LRRK2* has been studied in iPSCs-derived neurons [142]. LRRK2 plays a critical role in delayed mitophagy that leads to the accumulation of aberrant mitochondria [117,127]. An increased number of mitochondria with abnormal morphology, impaired functionality, and decreased membrane potential was detected in *LRRK2* mutant cells by Professor Walter’s group [117]. PD iPSCs-based models also revealed the progression of oxidative stress [120,121], damage of mitochondrial DNA, and energy imbalance [132]. The crucial role of mitochondrial impairments in PD pathogenesis was confirmed by experiments demonstrating a neuroprotective effect of coenzyme Q10, rapamycin, and the LRRK2 inhibitor GW5074 [121].

Different effects of the mutation in *LRRK2* on calcium dynamics in the endoplasmic reticulum have been obtained on iPSCs-based PD models [138]. It has been shown that incubation of dopaminergic neurons carrying the *LRRK2* G2019S mutation with thapsigargin (a blocker of SERCA) leads to a decrease in the length of neurites. This effect was not observed in healthy control iPSCs-derived neurons [138]. The SERCA inhibition-dependent neurite collapse has been confirmed in isogenic models [138]. Finally, *LRRK2* G2019S neurons were exposed to LRRK2 inhibitor MLi-2, as well as the use of antisense oligonucleotides that reduce *LRRK2* expression (targeting the mRNA of the *LRRK2* and preventing its translation) restore the neurite length in *LRRK2* G2019S dopaminergic neurons [138]. It has also been reported that *LRRK2* G2019S dopaminergic neurons incubated with thapsigargin showed a reduced calcium level in the endoplasmic reticulum, but an increased amount of calcium in the cytosol. In addition, there was an increased calcium uptake during depolarization [138]. The investigated pathological changes in calcium signaling could be rescued by antisense oligonucleotides to *LRRK2*. It is important to note that the dynamics of calcium in the endoplasmic reticulum is one of the key factors in the regulation of store-operated calcium entry. A decrease in the concentration of calcium in the endoplasmic reticulum leads to the activation of STIM proteins, which activate store-operated calcium channels. Although authors note a decrease in the expression of some genes (*STIM1*, *TRPC1*, and *ORA1*) responsible for the machinery of the store-operated calcium entry [138], its functional changes have not been studied yet what might be interesting for future investigations. iPSCs-based neurons with mutations in the *PINK1* and *PARK2* genes showed several types of mitochondrial dysfunction such as aberrant morphology, increased ROS levels, oxidative stress, decreased mitochondrial respiration, and impaired mitochondrial motility [41,51,133,134]. Notably, the mitochondrial dysfunctions were detected in differentiated neurons and absent in the fibroblast cultures and iPSCs obtained from patients with these mutations [41,51]. Mutations in the *DJ-1* gene are a rare cause of hereditary PD. Dopaminergic neurons with mutant *DJ-1* show mitochondrial dysfunction that leads to the accumulation of oxidized dopamine in PD, which could be prevented by the calcineurin inhibitor FK506 [129]. Thus, impaired calcium signaling may significantly contribute to dopamine metabolism. In addition to mitochondrial dysfunction, the functioning of voltage-gated L-, R-, T-, N-, and P/Q-type channels in PD is currently being discussed [140,143,144]. It was found that mRNA, which has a complex secondary structure in the 5’-untranslated region (UTR), is translated more efficiently in neurons with the G2019S mutation in *LRRK2*. This leads to increased expression of many genes involved in the regulation of calcium signaling, including voltage-gated calcium channel (VGCC) subunits, and consequently results in the greater calcium influx and intracellular calcium concentration. [136]. iPSCs-derived neurons with the *LRRK2* G2019S mutation also showed changes in neurite morphology and decreased calcium response under membrane depolarization conditions [125]. Impaired calcium signaling plays an important role in PD pathogenesis, which can be confirmed by the neuroprotective effects of small molecules and genetic approaches affecting calcium homeostasis machinery. For example, dopaminergic neurons obtained from a patient with a mutation in the *PARK2* gene were rescued from the rotenone-induced apoptosis by both the application of a VGCC antagonist benidipine and the knockdown of T-type calcium channels, thus demonstrating the involvement of voltage-gated calcium channels in PD pathology [140].

While most therapeutic strategies aim to prevent neuronal loss or protect vulnerable neurons, a potential alternative is to replace lost neurons to recover the neuronal network. A significant breakthrough was made by an article whose authors demonstrated the conversion of midbrain astrocytes to dopaminergic neurons, which provide axons to reconstruct the nigrostriatal circuit [145]. These data create new possibilities for the treatment of neurodegenerative diseases by replacing lost neurons.

## 7. Huntington’s Disease

Huntington’s disease (HD) is one of the inherited polyglutamine neurodegenerative disorders. In addition to HD, there are six types of spinocerebellar ataxias (SCA1, SCA2, SCA3, SCA6, SCA7, and SCA17), dentatorubral-pallidoluysian atrophy (DRPLA), and spinal and bulbar muscular atrophy (SBMA)/Kennedy’s disease [146]. These pathologies are associated with the apathogenic expansion of glutamine-encoding (CAG) repeats in the mutant genes, which leads to the production of mutant proteins containing an abnormally elongated polyQ tract. Huntington’s disease occurs when the number of CAG repeats in the huntingtin gene exceeds 35 [146]. The hallmark of the pathology is the aggregation of mutant huntingtin (mHTT). Furthermore, impairments in intracellular transport, autophagy, gene expression, calcium homeostasis, energy imbalance, and oxidative stress were reported in HD [147,148,149]. Interestingly, mHTT production is ubiquitous in human tissues, but HD is characterized by a unique pattern of neuronal death. The most remarkable changes found in HD are a selective loss of GABAergic striatal medium spiny neurons (MSNs), severe atrophy of the caudate nucleus and putamen, and atrophy of cortical neurons [150].

For the first time, iPSCs from a patient with HD were characterized in 2008 [151]. Later, differentiated neurons were obtained [152]. To use the iPSCs-based models correctly, it is necessary to know the stage of differentiation and maturity of cells when changes occur. The pronounced differences in gene expression between patient-specific NPC and isogenic control and the absence of such differences between HD-specific iPSCs and isogenic control have been reported [52]. In 2012, the HD iPSCs Consortium announced the creation and characterization of a panel of 14 iPSCs lines from HD patients and healthy donors [153]. The first iPSCs-based 3D model of HD was presented in 2018 [49]. Currently, iPSCs-based HD models with terminal differentiation into cortical [154] and GABAergic neurons [8,61,155,156,157,158], astrocytes [159,160], endothelial cells [71], and isogenic models [36,44] are studied (Table 3).

Transcriptome analysis of iPSCs-based HD models showed a lower expression of the genes involved in calcium signaling, including the genes that encode for NMDA and AMPA glutamate receptors, nicotinic acetylcholine receptor subunits, some voltage-gated calcium channel subunits, plasma membrane calcium ATPases, and various effectors such as calcium–calmodulin dependent protein kinase (CAMKII), CALM, and CREB [73]. Also, the expression of genes responsible for cell death was impaired [50]. Suppression of the gene expression encoding proteins involved in the metabolism and signal transduction pathways of glutamate and GABA was also noted [73]. On the other hand, increased expression of genes encoding store-operated calcium channels and the inositol triphosphate receptor was noted [73]. Functionally, increased store-operated calcium entry was observed in differentiated GABAergic MSNs in adult-onset [8,157] and juvenile HD models [61]. We showed that STIM2, as a key activator of store-operated calcium channels in neurons, may play a critical role in increased store-operated calcium entry in HD and be a target for potential HD drugs [61]. In addition, we noted increased calcium uptake through voltage-gated calcium channels in juvenile and adult-onset iPSCs-based HD models [61]. It is well-known that the length of the huntingtin’s polyglutamine tract directly correlates with the severity of HD and inversely correlates with the age of manifestation of the disease. At the moment, it has been noted that the amplitude of the store-operated calcium currents and, likely, calcium entry through VGCC did not depend on the length of the polyglutamine tract in the mutant huntingtin [61]. Nevertheless, it was noted that the length of the polyglutamine tract in the mutant huntingtin may impact mitochondrial dysfunction, the severity of DNA damage, and cell death, which was demonstrated on isogeneic NPC models of HD (81Q, 65Q, and 45Q) [44]. The most severe alterations were observed for NPC expressed huntingtin with a long polyglutamine tract (81Q).

HD is also characterized by mitochondria dysfunction: decreased mitochondrial density [156], the level of adenosine triphosphate (ATP), and decreased expression of glycolytic enzymes [161]. ATP levels in the neurons of HD patients can be restored by the addition of pyruvate; thus, pyruvate or other related metabolic supplements may have a therapeutic effect in HD [161]. A promising modulator of calcium signaling that can be used in the treatment of HD is EVP4593 compound. It has been shown that EVP4593 has neuroprotective properties [5,162], including in iPSCs-based models [8]. EVP4593 restored pathologically enhanced store-operated calcium entry [8,157], as well as reduced the level of huntingtin and the STIM2 with prolonged incubation (24 h) [61]. Also, the increased formation of lysosomes and autophagosomes have been reported in HD iPSCs-based models [8,155]. In turn, the compound EVP4593 normalized the number of lysosomes/autophagosomes in iPSCs-based HD neurons [8]. In HD-specific astrocytes, impaired calcium signaling (lengthening of spontaneous calcium oscillations) was noted [160], and impaired transcytosis was shown in endothelial cells [71]. Thus, using HD iPSCs-based models, it was possible to get closer to understanding the mechanisms of aberrant calcium signaling from the impaired expression of genes responsible for calcium signaling to the dysfunction of ion channels and mitochondria.

## 8. Spinocerebellar Ataxias

Spinocerebellar ataxias (SCAs) are a large (over 40 disorders) and diverse group of inherited neurodegenerative pathologies [163]. All of them are characterized by the progressive degeneration of the cerebellum, especially Purkinje cells [164]. Great success in obtaining a Purkinje cell culture differentiated from mouse [165,166] and human ESCs [47], and subsequently from iPSCs obtained from healthy donors and patients with SCA 42 [167] and SCA 6 [140], was achieved by the Japanese group of Yoshiki Sasai and Keiko Muguruma. These works showed that Purkinje cells can be obtained in 3D culture, which reproduces the microenvironment in vivo well [42,47,165,166,168,169]. However, the creation of iPSCs-based SCA models based on Purkinje cells remains a complicated task. Currently, only a few protocols for the differentiation of iPSCs into Purkinje cells are known [42,170,171,172,173]. There are few other iPSCs-based models of SCA; thus, obtaining further models remains an important task. The first iPSCs-based SCA model was investigated in 2011 [58]. Currently, the presented iPSCs-based SCA models are (Table 4): SCA 1 [174,175], SCA 2 [176,177,178], SCA 3 [58,59,60,178,179,180,181,182,183,184,185,186,187,188], SCA 6 [42,189], SCA 7 [190,191,192], SCA 14 [193], SCA 16 [194], SCA 36 [195], and SCA 42 [167]. More data accumulated on iPSCs-based models of different SCAs can be found in the review by Hommersom et al., 2021 [196].

It is known that SCA types 6 and 42 arise as a result of mutations in the *CACNA1A* and *CACNA1G* genes encoding voltage-gated P/Q- and T-type calcium channels, respectively [197]. Depending on the results of alternative splicing, the *CACNA1A* gene can encode not only the α1A subunit of the voltage-gated P/Q calcium channel, but also the α1ACT transcription factor. Ishida’s group focused on the dysregulation of α1ACT-dependent gene expression and the vulnerability of cells to oxidative stress [42]. In the work of Bavassano’s group, the authors investigated the expression and function of the *CACNA1A* gene products in a 2D culture of iPSCs-based neurons obtained from patients with SCA 6. Expression levels of *CACNA1A* encoding the α1A subunit were similar between SCA6 and control neurons, and no differences were found in the subcellular distribution of the Cav2.1 channel. Electrophysiological data showed that the obtained voltage-dependent calcium currents were sensitive to the selective blocker of P/Q channels ω-agatoxin IVA; however, no differences in the amplitudes of currents were found between normal and pathological conditions. The authors suggested that the absence of differences was due to use of the model, as the disease has a late age of manifestation, and the neurons obtained in culture were not mature enough to reveal the pathological phenotype [189]. Moreover, it can be suggested that the absence of differences is due to the use of GABA and glutamatergic neurons but not a Purkinje cell, which is well-known to be a primary target in ataxias.

The role of the p.Arg1715His mutation in the *CACNA1G* gene on neurogenesis has also been studied [167]. In that work, Purkinje cells differentiated from iPSCs of healthy donors and patients with SCA 42 did not demonstrate significant differences in either the morphology of the dendritic tree or the expression of markers of Purkinje cells (L7 and GRID2, a specific glutamate receptor of Purkinje cells); therefore, this mutation likely does not affect cell differentiation [167]. Unfortunately, there are currently no data on the effect of the p.Arg1715His mutation in the *CACNA1G* gene on the functioning of voltage-gated T-type calcium channels (Cav3.1) obtained from iPSCs-based models. However, it is known that this mutation affects the voltage sensor (segment S4) Cav3.1 and leads to a shift in the activation curve of mutant channels toward positive potentials compared to the control, without changing the maximum amplitude of currents through Cav3.1 [167].

Currently, interesting data demonstrating aberrant calcium signaling have been obtained in iPSCs-based models of polyglutamine ataxias, including SCA1, SCA2, SCA3, SCA7, and SCA17. According to our unpublished data, SCA1 and SCA17 are characterized by the disturbance of calcium signaling. We have shown an increase in store-operated calcium entry and calcium entry through VGCC in SCA17 GABAergic MSNs, which are also highly vulnerable in SCA17 as well as in HD (Vigont et al., unpublished data). In contrast, we observed a decrease in the store-operated calcium entry in SCA1 and no changes in the functioning of voltage-gated calcium channels in GABAergic neurons, which are not affected in SCA1 (Vigont et al., unpublished data). Unfortunately, at present, there is a lack of iPSCs-based models of SCA1 and SCA17, except for studies on differentiated neurons from patients with SCA1 [174,175] and the characterization of iPSCs from patients with SCA17 [198].

For SCA2 and SCA3, a change in the expression of the gene encoding subunits of glutamate receptors and other proteins involved in calcium signaling was shown. Significant suppression of gene expression and a decrease in the amount of glutamate receptor protein (GRIA4) and other participants in calcium signaling was noted in SCA3 when cells were incubated in a medium with glutamate [178]. In the presence of glutamate, neuronal death has also been increased; especially in SCA3, an increase in the concentration of cytosolic calcium occurs, and mitochondrial dysfunction is observed [178]. The use of glutamate receptor blockers prevented the release of calcium into the cytosol, leading to a mitigation of the pathological phenotype in SCA2 and SCA3. The most effective drug for arresting the pathological effects in SCA3 was dantrolene, which is known as a ryanodine receptor antagonist. This agent reduced cell death and the amount of calcium in the cytosol, as well as restored mitochondrial function [178]. Riluzole has been shown to be effective in the treatment of both SCA2 and SCA3. The less effective substances were MK801 and NBQX, known as NMDAR and AMPAR antagonists, respectively [178].

The production and accumulation of mutant polyglutamine proteins lead to the formation of intracellular aggregates. It was found that the formation of aggregates of mutant ataxin 3 is a calcium-dependent process. Treatment of iPSCs-based SCA3 neurons with glutamate enhanced the cleavage of ataxin 3, which was accompanied by the accumulation of its fragments and the formation of aggregates, leading to a decrease in the lifespan of neurons. In turn, blockers of calpain, which require calcium for its activation, had a neuroprotective effect and prevented the formation of aggregates [58]. Calpain-induced cleavage of ataxin 3 led to the formation of ataxin 3 fragments causing mitochondria fragmentation and cristae damage as well as disrupting the respiratory function of mitochondria, in addition to mitochondria degradation [179,181]. Notably, these alterations were not observed in both SCA3 fibroblasts [58] and NPS [188] but only in mature neurons.

Spinocerebellar ataxia type 7 (SCA7) is another polyglutamine disorder characterized by degeneration of the cerebellum and retina. Patients with SCA7 demonstrated atrophy of the cerebellar cortex and brainstem and significant loss of Purkinje cells. An important feature of SCA7 that distinguishes it from other spinocerebellar ataxias is retinal degeneration. As the disease progresses, dysfunction turns into complete blindness. Many clinical features of SCA7 were observed in mitochondrial diseases; therefore, it is interesting to study the functioning of mitochondria in patients with SCA7. NPCs obtained from iPSCs from patients with SCA7 showed a decrease in the length of the mitochondrial network. The strong changes were observed in a SCA7 patient with 70 glutamine residues in polyQ tract in ataxin7 (70Q) but only minor changes were detected in patients with a shorter mutant polyQ tract (50Q) and (65Q). It has been shown that the length of the mitochondrial network is reduced depending on the length of the polyglutamine tract in isogenic cell lines. In addition, NPCs producing ataxin7-113Q showed a marked decrease in the oxygen consumption rate (OCR) and a significant increase in the extracellular acidification rate (ECAR), which was possibly caused by a significant decrease in the NAD^+^ level in SCA7 NPC [191].

## 9. Amyotrophic Lateral Sclerosis

Amyotrophic lateral sclerosis (ALS) is one of the most severe neurodegenerative pathologies with the predominant death of motoneurons (MNs). Therefore, differentiated patient-specific motor neurons [64,199,200,201,202,203,204,205,206,207,208,209,210,211,212,213,214,215,216,217,218,219], as well as isogenic models [220,221,222,223], have been widely used to model this pathology since 2008. It is important to note that in the published papers, the proportion of MNs from the differentiated cells varies from 30% to 95% (Table 5). In Table 5, all the presented models are marked as “motoneurons.” Optimization of the protocols of iPSCs differentiation into MNs remains an important task. In addition to MNs, cortical neurons [205], astrocytes [202,224,225], and oligodendrocytes [226] were presented. More than 90% of cases of ALS are sporadic. Hereditary forms of ALS are associated mainly with mutations in the genes: *SOD1* (superoxide dismutase 1) [227], *TDP-43* or *TARDBP* (Tar DNA binding protein) [228], *C9ORF72* (chromosome 9 open reading frame 72) [229], and *FUS* (fused in sarcoma) [230]. Due to the use of iPSCs-based models, not only are hereditary forms of ALS currently being studied, but so are sporadic cases of ALS [205,209,215,216]. In iPSCs-based models of hereditary and sporadic ALS, changes in both gene expression and functional alterations associated with the mitochondria and calcium signaling were noted.

In iPSCs-based MNs with a mutation in the *C9ORF72* gene, an increase in the calcium influx to the cytosol after depolarization and a slow recovery up to physiological conditions was shown [219]. This study also demonstrated a decrease in the amount of calbindin, which may reduce calcium buffering capacity in the cytosol. [219]. *C9ORF72* ALS MNs were found to be more sensitive to glutamate treatment than control cells [204]. At the same time, glutamate-induced cell death was compensated for by treatment with blockers of glutamate receptors and calcium channels (MK-801, 10 µM; CNQX, 10 µM; nimodipine, 2 µM) [204]. Motor neurons with the *TARDBP* mutation showed high basal levels of intracellular calcium [218], as well as increased glutamate-induced calcium entry into the cytosol. Both MNs with mutations in *C9ORF72* and *TARDBP* have been shown to have a greater conductance of AMPA and NMDA receptors for calcium [219]. In addition, an increase in the expression of genes encoding kainate, AMPA, and NMDA receptors [217], as well as VGCC subunits [218], have been reported. No differences were found in the expression of genes encoding the Orai1, STIM1, and SERCA1 proteins involved in the store-operated calcium entry [218]. At the same time, a decrease in the thapsigargin-induced release of calcium from the ER with mutations in the *SOD1*, *FUS*, and *TDP43* genes was noted [218]. Motor neurons with a mutation in *SOD1* were characterized by changes in gene expression and functional disorders associated with the mitochondria. There was a change in morphology, a decrease in mobility, and an increase in the density of mitochondria [224]. Some mitochondrial disorders are also characteristic of MNs with mutations in *C9ORF72* and *TARDBP*: a decrease in the mitochondrial membrane potential [212], a decrease in the calcium buffering capacity [219], and dysfunction of mitochondrial uniporters [219,221]. MNs with the *FUS* mutation are characterized by the absence of morphological changes in mitochondria; however, a decrease in their motility was noted. Moreover, the severity of the impairment increases with the maturation of neurons [223]. For sporadic cases of ALS, changes in gene expression associated with mitochondrial function were noted [209]. Motor neurons with a mutation in *SOD1* were also characterized by oxidative and ER stress, hyperexcitability of neurons [221], and a decreased level of calcium-binding proteins CDH23 and CALU [220].

The data analysis allows us to conclude that the aberrant calcium signaling observed in ALS highly varies depending on the gene in which the mutation occurs [219,221] (Table 5).

## 10. Other Neurological Diseases

### 10.1. Autism Spectrum Disorders

Autism spectrum disorders (ASD) are a heterogeneous group of pathologies associated with impairments in the development of the nervous system. ASD have an unclear etiology: A fairly large number of gene mutations that can be associated with autism has been identified. Among them are the genes responsible for the functioning of voltage-gated calcium channels (VGCC): *CACNA1A, CACNA1B, CACNA1C, CACNA1D, CACNA1E, CACNA1F, CACNA1G, CACNA1H*, and *CACNA1I* as well as accessory subunits *CACNB2, CACNA2D3*, and *CACNA2D4* [231].

Timothy’s syndrome is a prominent example of ASD associated with mutations in voltage-gated channels. This pathology results from a mutation in the *CACNA1C* gene encoding voltage-gated L-type calcium channels (Cav1.2) [232]. The mutation is localized in the S6 segment and leads to impaired inactivation of the Cav1.2 channels [232]. ASD are characterized mostly by the dysfunction of cortical neurons; therefore, ASD including Timothy’s syndrome are commonly modeled by iPSCs-based cortical neurons. Recent data on modeling Timothy’s syndrome on patient-specific neurons are presented in detail in a review [233]. iPSCs-based neurons from patients with Timothy’s syndrome were characterized by a decrease in the dendritic tree [234]. Using calcium imaging (Fura 2AM), a significant increase in the depolarization-induced calcium influx, which was sensitive to the L-type calcium channel blocker nimodipine, has been shown in iPSCs-based neurons of patients with Timothy’s syndrome (Table 6). Also, using Illumina microarrays, changes in the expression of 223 genes in patients with Timothy’s syndrome were shown, some of which are responsible for calcium signaling [235]. In addition, it has been shown that VGCC in Timothy’s syndrome are involved in neuronal differentiation and developmental regulation [236].

It is also important to note that disturbances in the functioning of voltage-gated calcium channels can be caused not only by mutations of the genes encoding them but also by the dysfunction of regulatory proteins. For example, neurexins are transmembrane, predominantly presynaptic proteins involved in the regulation of different calcium channels, including VGCC. It has been shown that neurons with a mutation in the neurexin gene (NRXN1α ^+/−^) obtained from iPSCs of patients with autism exhibit changed VGCC functioning with an increased frequency, duration, and amplitude of calcium oscillations [238].

### 10.2. Leigh’s Syndrome

Leigh’s syndrome (LS) is the most frequent mitochondrial disorder in infants and is characterized by neurodegeneration and astrogliosis in the basal ganglia or brainstem. There are currently no drugs or effective treatments for this disease, in part due to a lack of relevant models. More recently, an iPSCs-based model of LS carrying the mtDNA m.13513G mutation has been created (Table 6). Characterization of mitochondria, electrophysiological analysis, and calcium imaging in iPSCs-based neurons were carried out. Impairment of oxidative phosphorylation in the neurons of LS patients was clearly demonstrated. This is the first report of electrophysiological studies performed on iPSCs-derived neurons carrying the mtDNA mutation, which showed that affected neurons exhibit mitochondrial dysfunction along with decreased calcium buffering capacity. This can lead to an increase in cytoplasmic calcium concentration and subsequent cell death seen in patients [237].

## 11. Conclusions

In summation, we can say that iPSCs-based models became important instruments for studying the mechanisms underlying neurodegenerative pathologies and screening potential neuroprotective drugs. In particular, rare pathologies such as HD, SCAs, LS, and others have become available for research. Moreover, iPSCs-based models opened up the possibility of studying sporadic forms of neurodegenerative diseases, which are most common in AD, PD, and ALS. In general, studies carried out using iPSCs-based models reproduce previously obtained data in animal and cellular models, but at the same time significantly deepen our understanding of the molecular mechanisms of pathogenesis. Although the molecular mechanisms of the pathogenesis of various neurodegenerative diseases may differ, aberrant calcium signaling is characteristic of most of them. The most common abnormalities of calcium signaling in neurodegenerative pathologies include altered functioning of calcium-permeable channels, changes in levels of calcium-binding proteins, and mitochondrial dysfunctions. A stable increase in the concentration of calcium in the cytosol may lead to the launch of the apoptotic cascade and cell death. To prevent cell death, various compensatory mechanisms regulate the dynamics of calcium in the cell. For example, excess calcium can be accumulated in intracellular calcium stores (mitochondria and endoplasmic reticulum) or buffered by calcium-binding proteins. Thus, the often observed alterations in the expression of the genes encoding calcium-binding proteins may be separated from disease causes but represent a compensatory mechanism seeking to prevent cell death.

The collected data allow us to suggest a central role of calcium signaling disturbances in neurodegenerative processes and establish the components of calcium signaling machinery to be a promising target for medical treatment. Summarizing the data, we can also suggest that the pathological increase of calcium uptake along with the greater calcium release from intracellular stores is more dangerous for cells than pathological attenuation of calcium signaling pathways. So, screening for new drugs in general should focus on finding the novel inhibitors of calcium channels and transporters. Additionally, iPSCs-based models open up studies of the neuronal specificity of the pathological processes and may shed light on the problem of the selective vulnerability of neurons in distinct pathologies. We would like to believe that in the near future the use of iPSCs-based technologies will not only clarify the molecular mechanisms of neurodegenerative processes but also contribute to the development of novel and very specific neuroprotective drugs.

## Figures and Tables

**Table 1:** Alterations in calcium signaling in iPSCs-based models of Alzheimer’s disease.

Alzheimer’s Disease			
The most vulnerable areas of the brain		iPSCs-based models	
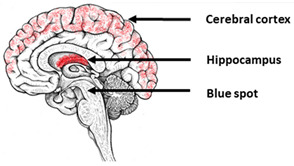		Patient-specific models	
		Neurons	[37,46,48,97,99,100,101,102,103,104]
		Cortical neurons	[105,106]
		Cholinergic neurons	[107]
		Astrocytes	[105,108]
		Endothelial cells	[72,109]
		Microglia	[67,69]
		Isogenic models	
		Neurons	[37,38,39,98]
Mutant genes	Cell type	Disturbance associated with calcium signaling	
*PSEN1*	Neurons	Increased ROS production, impaired energy status and mitochondrial potential, impaired autophagy, ER stress and increased calcium release from the ER	[104]
	Astrocytes	Disturbed calcium release from the ER, increased ROS production	[108]
*PSEN2*	Neurons	Increased amplitude of spontaneous calcium oscillations and their desynchronization, hyperactivation of neurons	[99]
*APP*	Astrocytes	ER stress, increased ROS production	[105]
Sporadic forms	Neurons	Mitochondrial dysfunction, increased ROS production, increased levels of oxidative phosphorylation chain complexes	[103]

**Table 2:** Alterations in calcium signaling in iPSCs-based models of Parkinson’s disease.

Parkinson’s Disease			
The most vulnerable areas of the brain		iPSCs-based models	
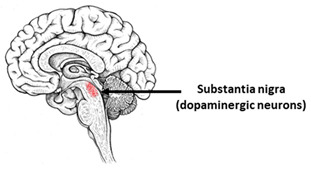		Patient-specific models	
		NESC	[117]
		Dopaminergic neurons (DAn)	[51,118,119,120,121,122,123,124,125,126,127,128,129,130,131,132,133,134,135,136,138]
		Cortical neurons	[137]
		Astrocytes	[66]
		Microglia	[68]
		Isogenic models	
		DAn	[41,45]
		Cortical neurons	[43]
Mutant genes	Cell type	Disturbance associated with calcium signaling	
*LRRK2*	NESC	Mitochondrial dysfunction, impaired mitophagy	[117]
	DAn	Enhanced expression of genes involved in calcium signaling, as well as an increase in calcium influx, mitochondrial dysfunction, mitochondrial DNA damage, oxidative stress, disrupted calcium dynamics in the ER	[120,121,124,125,127,131,132,136,138]
*SNCA*	Cortical neurons	Mitochondrial dysfunction, oxidative stress	[137]
	DAn	Mitochondrial dysfunction, ER stress, impaired calcium homeostasis and ATP production	[43,119,130,131,135,139]
*PINK1*	DAn	Mitochondrial dysfunction, oxidative stress, slowed utilization of damaged mitochondria	[133]
*PARK2*	DAn	Mitochondrial dysfunction, oxidative stress, dysfunction of voltage-gated T-type calcium channels	[41,51,134,140]
*DJ-1*	DAn	Oxidative stress	[129]

**Table 3:** Alterations in calcium signaling in iPSCs-based models of Huntington’s disease.

Huntington’s Disease			
The most vulnerable areas of the brain		iPSCs-based models	
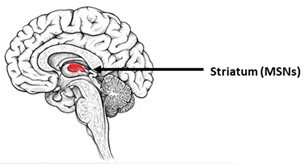		Patient-specific models	
		iPSCs	[50]
		NPC	[52,152,153,161]
		Cortical neurons	[154]
		GABAergic neurons	[8,61,155,156,157,158]
		Astrocytes	[159,160]
		Endothelial cells	[71]
		Mixed cultures	[73]
		Isogenic models	
		NPC	[44]
		GABAergic neurons	[36]
Mutant genes	Cell type	Disturbance associated with calcium signaling	
*HTT*	iPSCs	Impaired expression of the genes responsible for cell death	[50]
	Mixed cultures	Impaired expression of genes responsible for calcium signaling	[73]
	GABAergic neurons	Increased store-operated calcium entry and calcium currents through voltage-gated calcium channels in low-repet and juvenile models of Huntington’s disease	[8,61,157]
	GABAergic neurons	Increased mitochondrial density	[156]
	GABAergic neurons	Dysfunction of lysosomes and autophagy	[8,155]
	NPC	Mitochondrial dysfunction, bioenergetic defects	[44,161]

**Table 4:** Alterations in calcium signaling in iPSCs-based models of spinocerebellar ataxias.

Spinocerebellar Ataxias			
The most vulnerable areas of the brain		iPSCs-based models	
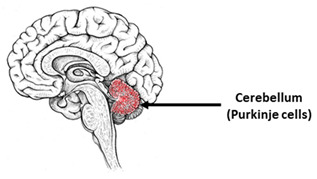		Patient-specific models	
		iPSCs	[193]
		NPC	[176,177]
		Neurons	[58,59,60,174,175,180,182,183,184,185,186,187,188,190,192]
		Cortical neurons	[179,181,194]
		Motoneurons (MNs)	[195]
		GABAergic and glutamatergic neurons	[189]
		Purkinje cells	[167,170,171,172,173]
		Mixed cultures	[178]
		Isogenic models	
		NPC	[191]
		Purkinje cells	[42]
Mutant genes	Cell type	Disturbance associated with calcium signaling	
*ATXN1*(SCA1)	GABAergic neurons	Decreased store-operated calcium entry, no changes in the functioning of voltage-gated calcium channels	Vigont et al., unpublished data
*ATXN2* & *3*(SCA2 & 3)	Mixed cultures	Altered expression of glutamate receptor subunits and other participants in calcium signaling. Increased calcium concentration in the cytosol. Distorted mitochondrial microstructures and mitochondrial dysfunction	[178]
*ATXN3*(SCA3)	Cortical neurons	Mitochondrial fragmentation and cristae alterations leading to decreased capacity of mitochondrial respiration, impaired mitochondrial degradation. Enhanced calpains-activated ataxin 3 cleavage and produce ataxin 3 fragments.	[58,179,181]
*CACNA1A* (SCA6)	GABAergic& glutamatergic neurons	No differences in both CACNA1A expression and amplitudes of voltage-gated calcium currents	[189]
*ATXN7*(SCA7)	NPC	Decreased mitochondrial network length. Mitochondrial dysfunction, reduced oxygen consumption rate and increased extracellular acidification rate	[191]
*TBP*(SCA17)	GABAergic neurons	Increased store-operated calcium entry and voltage-gated calcium uptake	Vigont et al., unpublished data

**Table 5:** Alterations in calcium signaling in iPSCs-based models of amyotrophic lateral sclerosis.

Amyotrophic Lateral Sclerosis			
The most vulnerable areas of the brain		iPSCs-based models	
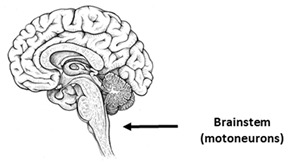		Patient-specific models	
		Motoneurons (MNs)	[64,199,200,201,202,203,204,205,206,207,208,209,210,211,212,213,214,215,216,217,218,219]
		Cortical neurons	[205]
		Astrocytes	[202,224,225]
		Oligodendrocyte	[226]
		Isogenic models	
		Motoneurons (MNs)	[43]
Mutant genes	Cell type	Disturbance associated with calcium signaling	
*C9orf72*	MNs	Transcriptional changes in levels of the mitochondrial transporter, increased expression of genes encoding glutamate receptors and VGCC. Increased glutamate excitotoxicity *C9ORF72* iPSN cultures. Disrupted mitochondrial calcium buffering capacity, low calbindin levels	[204,217,218,219,221]
*TARDBP*	MNs	Increased expression of genes encoding glutamate receptors. Elevated basal intracellular calcium levels. Increased conductance for calcium AMPA and NMDA receptors. Imbalance in mitochondrial calcium buffering capacity	[217,218,219]
*SOD1*	MNs	Transcriptional and functional changes: defects in mitochondrial transport, morphology and motility, increase in mitochondrial density, oxidative and ER-related stress. Reduced level of calcium-binding proteins.	[220,221]
*FUS*	MNs	No morphological changes in the mitochondria, decreased mitochondrial motility	[223]
Sporadic form	MNs	Deregulated expression of the genes associated with mitochondrial functioning	[209]

**Table 6:** Alterations in calcium signaling in iPSCs-based models of other neurological diseases.

Other Neurological Pathologies			
The most vulnerable areas of the brain		iPSCs-based models	
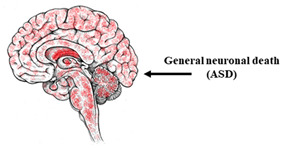		Patient-specific models	
		NPC (LS)	[237]
		Neurons (ASD)	[234,235,236]
		Cortical neurons (ASD)	[238]
Mutant genes	Cell type	Disturbance associated with calcium signaling	
Genes encoding VGCC subunits (ASD)	Neurons	Altered expression of genes responsible for calcium signaling. Dysfunction of voltage-gated channels. Increased nimodipine sensitive calcium influx after depolarization.	[234,235]
mtDNA m.13513G (LS)	NPC	Mitochondrial dysfunction, decreased calcium buffering capacity	[237]

## Data Availability

Not applicable.
